# Department of Therapeutics

**Published:** 1896-02

**Authors:** 


### Current Medical Literature.

### DEPARTMENT OF THERAPEUTICS.

UNDER THE CHARGE OF DAVID CERNA, M. D., PH. D.,
Demonstrator of Physiology and Lecturer on the History of Medicine in the Medical Department of the University of Texas, Galveston.

USES OF COCAINE AS A MYDRIATIC.—When using cocaine to dilate the pupil for the examination of the posterior segment of the eye with the ophthalmoscope, or the determination of the refraction in persons past fifty years of age, Jackson (*The Philadelphia Polyclinic, Dec. 28, 1895*) finds it important, by especial care, to prevent drying of the cornea and disturbance of its epithelium. Such drying and disturbance of the corneal surface will cause an irregular astigmatism that will cloud the ophthalmoscopic view of the fundus, or greatly impair the subjective test for glasses, by lowering the visual acuteness. It is easily prevented by using only so much cocaine as is necessary (one drop of a four per cent solution); and after its instillation keeping the eye closed most of the time for fifteen or twenty minutes.

THE THERAPEUTIC USES OF MESCAL BUTTONS (*Anhalonium Lewinii*),—Interesting studies of the physiological actions and therapeutic uses of *anhalonium lawinii* have been made by Prentis and Morgan (*Therapeutic Gazette, Sept. 15, 1895, and Jan. 15, 1896*). They find that this drug produces cerebral stimulation, accompanied with various visions, and sometimes slowness of thought, articulate aphasia, and marked delusion; dilatation of the pupil, lasting from twelve to twenty-four hours, accompanied with loss of accommodation and consequent disturbance of vision; more or less muscular depression; partial anæsthesia of the skin; a slow and weak pulse at first; this effect soon passes off; nausea and vomiting; sleeplessness; loss of sense of time, but no constant effect upon the bowels, skin, temperature, and the secretions, was found. The authors suggest the use of the drug in the following disorders: Nervousness, nervous headache, nervous irritable cough, abdominal pain due to colic or griping of the intestines, hysterical manifestations, and in other similar affections where an antispasmodic is indicated; as a cerebral stimulant in depressed conditions of the mind—hypochondriasis, melancholia, and allied conditions; as a substitute for opium and chloral in conditions of great nervous irritability and restlessness, active delirium and mania, and in insomnia caused by pain; in color-blindess. A tincture (10 per cent strength) and a fluid (100 per cent strength) of the remedy are recommended in doses of one to two teaspoonfuls (4 to 8 grammes) of the former, and ten to fifteen drops (0.50 to 1.00 gramme) of the latter preparation. The powder of the drug may be administered in doses of seven to fifteen grains (0.50 to 1.00 gramme). The active principles of this plant, anhalin, said to be alkaloid, is announced, the physiological actions of which are now being studied by the authors.

---

FORMALIN IN CUTANIOUS AFFECTIONS.—At a meeting of the Berlin Dermatological Association formalin was recommended in the treatment of hidrosis, in a 0.10 to 0.20 per cent solution; as also a mixture of formalin and tannin, which has proved very successful in severe cases of hidrosis and bromihidrosis.—*Journal of the American Medical Association, Jan, 25, 1896.*

---

THE ACTIONS OF THYROANTITOXIN.—Fränkel (*Med. Weekly, III. p. 672, 1895,—American Medico-Surgical Bulletin, Jan. 11, 1896*) has found that the albuminoid substances precipitated by acetic acid from a decoction of dried thyroid glands possess no especial properties, and that the really active substance contained in the thyroid gland, remains in the liquid obtained after separation of the albuminoid substances. From this liquid he has obtained a crystalline, very hygroscopic body, which gives the majority of the characteristic reactions of alkaloids. Its chemical formula is said to be $C_1\ H_{11}\ N_3\ O_5$, and it appears to be a derivative from the guanidine series. It is soluble in water and in alcohol, the aqueous solution being neutral or faintly alkaline in reaction and yielding a precipitate on the addition of lead acetate or acetic acid. Intravenous injections of this substance, for which he proposes the name of *thyroantitoxin*, determined in animals the quickening of the pulse, which is characteristic of injections of thyroid extract, and checks the convulsions in thyrodectomized animals. In animals into which this antitoxin is injected immediately after the extirpation of the thyroid body, no convulsions occur. The actions of this substance, however, is confined to the suppression of convulsions, and does not extend to the other symptoms of cachexia strumipriva. The animals ultimately die, because the antitoxic function of the thyroid gland is not supplied by thyroantitoxin.

---

THE MEDICAL TREATMENT OF GALL-STONES.—The treatment of gall-stones is editorially treated in a masterly manner by the *Therapeutic Gazette, January 16, 1896*. Among other considerations it is held that the catarrhal condition, associated with marked bacterial infection, as may be evidenced by some febrile movement, is best controlled by the use of turpentine, chloroform or ether, given internally, and accompanied by the application externally of hot poultices to the hepatic area. These poultices may or may not be fortified by mustard, and when removed, should be replaced by a warm pad to prevent any chilling of the surface of the body. Of the internal remedies just named, turpentine is the most useful, since it liquifies mucus, aids the flow of bile, and is thought by some physicians to cause the expulsion of the stone by stimulating the walls of the ducts, and that it dissolves the stone. Balfe states that it is best given as follows:

℞

Oı. terebintbinæ....................m. v.
Syrup acaciæ ... ............ ....f. ℥ss.
Sodii sulph. carbolat.,..............gr. xx.
Spirit. ætheris composit.,....... ...m. xv,
Aquæ menthæ piperitæ,.........q. s. f. ℥j.

M. Sig. To be taken twice or thrice daily.

We would prefer adding compound spirit of lavender instead of peppermint water. If the mixture can not be retained by the stomach, the turpentine may be given in capsule, and followed by a draught of milk. Finally, a most important factor in the prevention of gall-stone formation in susceptible persons is the avoidence of exposure and wet, and, if possible, a residence in a sunny climate during the winter months.

PILOCARPINE IN DISEASES OF THE LYMPH GLANDS.—Waldstein (*Berliner Klinische Wochenschrift, No. 32, 1895.—University Medical Magazine, January, 1896*), having demonstrated that subcutaneous injections of pilocarpine were followed by a distinct increase in the number of leucocytes, and that the drug exerted no local irritation, determined to try the remedy in lymphatic enlargements. The results were highly successful in the inflammatory enlargements of the lymph-glands accompanying scarlet fever and measles in children. One part of pilocarpine hydrochlorate was dissolved in 400 parts of water, and of this solution, sixteen minims (one twenty-fourth of a grain) constitnted a dose. This amount, the author claims, can be given daily for a week or ten days to children three years old, and seldom causes salivation or sweating. In some cases the daily amount for children of three years or under may be increased to one-twelfth of a grain. In a case of multiple lymphoma, and in a case of lupus of the hand the remedy gave good results. In the latter, however, it was impossible to predict a positive cure. Pilocarpine in daily doses of one-fiftieth to one-twelfth of a grain was also used in seven cases of streptococcus angina in children ranging from three to eighteen years. All recovered, and in only one case did the drug cause sweating and salivation.

